# Human Epididymis Protein 4 (HE4) Reference Limits in Polish Population of Healthy Women, Pregnant Women, and Women with Benign Ovarian Tumors

**DOI:** 10.1155/2019/3890906

**Published:** 2019-08-22

**Authors:** Emilia Gasiorowska, Tomasz Kluz, Dawid Lipski, Wojciech Warchoł, Andrzej Tykarski, Ewa Nowak-Markwitz

**Affiliations:** ^1^Department of Gynecology, Obstetrics and Gynecologic Oncology, Division of Gynecologic Oncology, Poznan University of Medical Sciences, Poland; ^2^Department of Obstetrics and Gynecology, Fryderyk Chopin University Hospital No 1, Rzeszów University, Poland; ^3^Department of Hypertensiology, Angiology and Internal Medicine, Poznan University of Medical Sciences, Poland; ^4^Department of Biophysics, Poznan University of Medical Sciences, Poland

## Abstract

**Objectives:**

Defining precisely the normal range of HE4 protein is crucial for the proper interpretation of tumor marker results and for a more efficient diagnosis of ovarian malignancy. The aim of our study was to evaluate a reference limit of HE4 protein in a population to promote and facilitate the common use of HE4 protein assays. We also tried to identify potential association of HE4 levels with other conditions such as smoking, age, BMI, and creatinine levels.

**Methods:**

Blood samples were collected from 617 patients divided into three groups: healthy, pregnant, and with benign ovarian tumors. Serum HE4 concentrations were measured following a standard procedure. HE4 reference ranges for each group and association of HE4 levels with BMI, creatinine, and smoking were investigated.

**Results:**

HE4 reference limit for healthy patients equals 85 pmol/l, which becomes 73 pmol/l and 93 pmol/l for pre and postmenopausal subgroups, respectively. There is a statistically significant correlation between HE4 serum level and smoking (*p* = 0.000001) and there is no correlation with creatinine. But if we take into account age and smoking, in multivariate analysis, there is a correlation. For pregnant, the upper limit values of normal HE4 levels are 55 pmol/l (median = 40 pmol/l), 80 pmol/l (median = 43 pmol/l), and 106 pmol/l (median = 53 pmol/l) for the first, second, and third trimesters, respectively.

**Conclusions:**

HE4 protein value strongly depends on the patient's age and smoking. The serum concentration of HE4 marker increases with the duration of pregnancy. Understanding the normal range of HE4 protein enables the correct interpretation of marker measurements. This may result in an earlier and more effective diagnosis of ovarian cancer.

## 1. Introduction

Human epididymis protein 4 (HE4) is a glycoprotein originally found in the epithelial cells of the human epididymis. It belongs to the family of serine protease inhibitors (WAP domain proteins) and plays a role in carcinogenesis, tumor progression, and metastasis [1, 2]. Since 2011, the HE4 protein has become an integral diagnostic tool for ovarian cancer [1]. Its levels, which are a malignancy risk predictor in women with adnexal mass, are measured in single tests, in series of tests, or as part of a Risk of Ovarian Malignancy Algorithm (ROMA) [2]. It is necessary to know how to interpret single and serial measurements of HE4 protein concentration. However, studies to date have not provided a universal reference range of HE4 levels for healthy women from different populations. HE4 norms used by different laboratories vary, and it is advisable to establish consistent norms for specific populations. The norm for premenopausal women of <70 pmol/l and for postmenopausal women of <140 pmol/l is the most commonly defined. Precisely defining the normal range is crucial for proper interpretation of tumor marker results and for a more efficient diagnosis of ovarian malignancy. Moreover, defining the normal range of HE4 levels in pregnant women would be even more useful because the cases of pregnancy with adnexal masses represent one of the most important obstetric clinical issues that leads to additional hospitalizations for pregnant women. Knowing a reference limit would bring both medical and economic benefits, as the eventual maternal and fetal complications, and the financial costs, of unnecessary surgical interventions could be avoided.

In order to promote and facilitate the use of HE4 protein assays, we attempted to define a normal range of HE4 protein in Polish populations of healthy women, pregnant women, and women with benign ovarian tumors. We also attempted to identify any potential associations between HE4 levels and parameters such as smoking, age, BMI, and creatinine levels.

## 2. Materials and Methods

We defined the inclusion criteria for our study based on the criteria in the literature that documents the associations between the HE4 marker and neoplasms of the female reproductive tract [1], the breasts [3], the pancreas [4], and the respiratory system [5]. And we also considered associations with chronic kidney disease [6] and any other conditions that are known to influence HE4 levels. Our inclusion criteria were as follows: absence of pathological changes in the reproductive organs confirmed by gynecological and ultrasound examinations; no history of chronic or acute infection in the past 6 months confirmed by CRP < 5; no history of blood transfusion or surgical treatment in the past 6 months; creatinine level < 1 mg/dl; and no history of acute or chronic diseases of the kidney, liver, or respiratory system.

A total of 617 patients participated in the study. The Local Bioethics Committee approved the study (reference number 838/14), and informed consent was obtained from all patients participating in the study.

Our study population was divided into three groups, and serum samples were collected from each participant. Blood samples were obtained by venous puncture, centrifuged, and stored at −80°C until the analyses were performed. The HE4 assays were performed with the use of electrochemiluminescence Elecsys® ECLIA by ROCHE. The concentrations were analyzed by the COBAS apparatus, used according to the manufacturer's instructions.

The first of our groups was comprised of patients admitted to the Gynecologic Oncology Department of Poznan University of Medical Sciences for surgical treatment of pelvic organ prolapse and urinary incontinence, in addition to the patients attending the Department of Hypertensiology, Angiology, and Internal Medicine of Poznan University of Medical Sciences for prophylactic checkups. The second group was comprised of patients of the Gynecologic Oncology Department of Poznan University of Medical Sciences who had qualified for surgical treatment of benign ovarian tumors followed by histopathological diagnosis. The third group was comprised of pregnant women attending routine control examinations at the Department of Obstetrics and Gynecology of Rzeszów University.

Patients with benign ovarian tumors were included in the study because the HE4 protein is registered in the differential diagnosis of the women's ovarian tumors, and not as part of a screening test in healthy patients. Therefore, it was very important to determine the HE4 limit value not only for healthy patients but also for patients with benign tumors, to help differentiate those patients from ovarian cancer patients.

The first group comprised of 265 healthy women aged 17 to 78 who exhibited no changes in the adnexa. We subdivided this first group into pre (*n* = 157) and postmenopausal groups (*n* = 108), with median ages of 38 years (range 17-54) and 59 years (range 45-78), respectively (Table 1).

The second group comprised of 195 patients with a benign ovarian tumor diagnosed by histopathological examination, of which 139 patients were premenopausal (median age 37, range 16-53) and 56 patients were postmenopausal (median age 60, range 49-83). Patients diagnosed with ovarian cancer intraoperatively were not included in the study.

The third group comprised of 157 healthy women with a median age of 30 (range 18-46), with a single pregnancy and with no concomitant diseases. This group was subdivided into trimesters, with 57 women in the first trimester, 24 in the second, and 76 in the third (Table 1).

After serum HE4 concentrations were assayed, we analyzed the levels considering patient age and classified the findings by age decades.

We established a 95% confidence interval for the normal range of HE4 with a 95% confidence limit (*CL95*%). This way we developed a separate reference range for each group, namely, for the pre and postmenopausal groups, as well as for the healthy group, and again separately for the healthy group that included the benign tumor patients.

We also analysed the data for any association between HE4 levels and the patients' BMI, creatinine, and smoking. The HE4 levels per trimester of pregnancy were analyzed, and a reference range was defined for each trimester.

The statistical analysis for our study was performed by the Biophysics Department at the Poznan University of Medical Sciences using Statistica 10 software from StatSoft; and *p* < 0.05 indicated a statistically significant difference. The Shapiro-Wilk test, the Spearman's test, the Kruskal-Wallis test, and the Mann–Whitney test were used.

## 3. Results

We observed that the HE4 levels rose correspondingly with the rising age of patients for both the healthy and the benign tumor groups (Table 2).

HE4 median and range were established as reference norms for each group. The 95% cutoff value for the healthy patients was 85 pmol/l, and for the pre and postmenopausal subgroups, it was 73 pmol/l and 93 pmol/l, respectively. After extending the healthy group to include the benign adnexal mass group, the new HE4 limit value was 91.5 pmol/l, and correspondingly, for the pre and postmenopausal women, it was 79 pmol/l and 117 pmol/l, respectively (Table 3).

**Figure 1:**
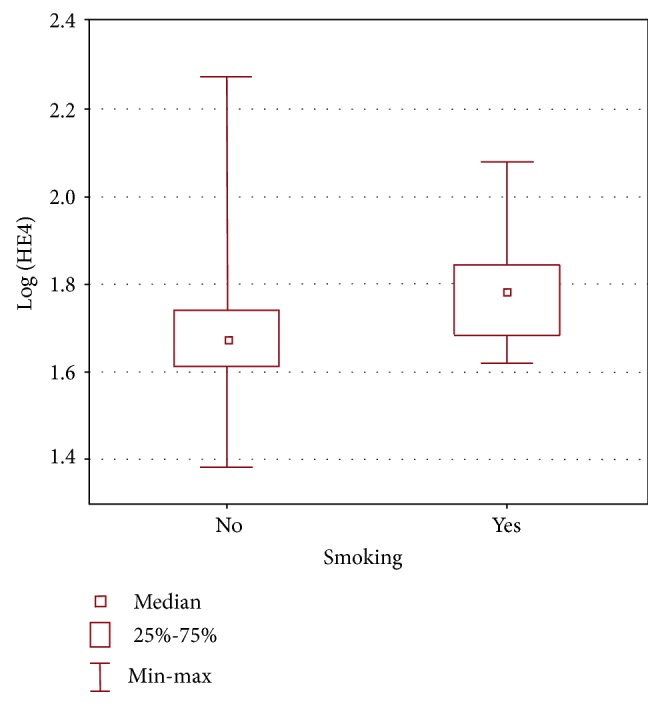
Range of HE4 concentrations in smokers and nonsmokers.

The mean BMI was 25 kg/m^2^ (range 17-43 kg/m^2^). The average creatinine concentration was 0.73 mg/dl (range 0.34-1.08 mg/dl). 27% of the patients smoked and 73% did not smoke. Among the members of the premenopausal group, 36% women smoked and that of the postmenopausal group, 17% smoked. When we analyzed the associations between the HE4 concentrations and the women's BMI, creatinine, and smoking, we found no correlation for BMI or creatinine. However, we did find a positive correlation between HE4 levels and smoking (*p* = 0.001), which is presented in Table 4 and Figure 1. Although, there is no statistically significant relationship between HE4 and creatinine, multivariate analysis reveals a statistically significant relationship between them, if we take into account age and smoking (Table 5).

The median HE4 concentration of the pregnant women was 46.0 pmol/l (range 15-117 pmol/l). After adjusting for the gestation age, we established that the HE4 confidence limits were 55 pmol/l (median 40 pmol/l), 80 pmol/l (median 43 pmol/l), and 106 pmol/l (median 53 pmol/l) for the first, second, and third trimesters, respectively (Table 6).

**Figure 2:**
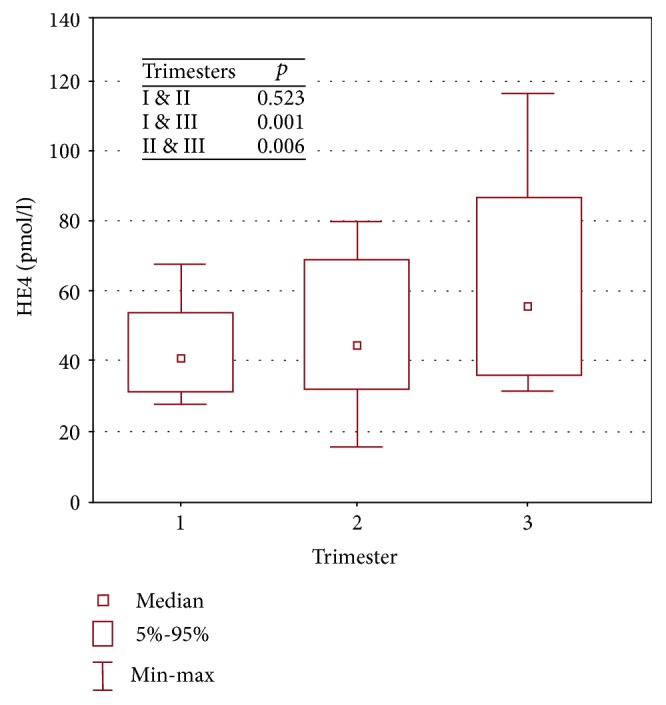
Serum HE4 levels by pregnancy trimester.

The differences in HE4 concentrations were statistically significant when separately comparing the results for the first and second trimesters with those of the third trimester (I and III, II and III) (Figure 2).

Despite the absence of any statistically significant difference between the first and second trimester results, analysis clearly showed that as pregnancy progresses, so does the concentration of the HE4 protein.

## 4. Discussion

### 4.1. HE4 and AGE

It is very important for clinical practitioners to have a clear and universal reference range for the HE4 protein at their disposal and to be aware of the factors that may influence its concentration levels.

The correlation we observed between rising HE4 concentration and advancing age has particular, and cautionary, implications when making clinical decisions based on HE4 levels in a patient when these levels are monitored over the course of many years.

Our study's findings, which are in line with those of the literature [7], show that the range of possible differences in HE4 levels that correspond with rising age is of particular importance for clinicians. For instance, when comparing the HE4 levels of women patients at different ages using 20 years old as the baseline, the levels were 9% higher by the age of 40 and 63% higher by the age of 70.

Moore et al. report a median HE4 level of 43.5 pmol/l for 40-year-olds and 66.9 pmol/l for 70-year-old women patients [8]. Our study showed the comparable results of 48 pmol/l and 62 pmol/l for women at age 40 years and 70 years, respectively.

Such findings point to potential problems with interpretation of HE4 assays, particularly when different results arise from examinations carried out over several years, such as in cases of monitoring of at-risk patients whose rising HE4 concentration may be attributed to their advancing age alone, irrespectively of any potential malignancy. For this reason, we postulate that doctors should know the positive correlations of HE4 protein levels with age, as well as the reference values for each age group.

Studies report varying reference ranges for HE4 as a consequence of the differences between the various study populations. However, overall, those studies' results are comparable. Moore et al. found the HE4 reference limit for pre and postmenopausal American women to be 89 pmol/l and 128 pmol/l, respectively [8]. In turn, our study, based on a Central European population, and the same groupings, found the reference limits to be 73 pmol/l and 93 pmol/l, respectively. Interestingly, when we include women with benign adnexal changes who are otherwise healthy, our reference limits became closer to Moore's American findings and became 89 pmol/l and 117 pmol/l, respectively.

Another study, published in 2015, attempted to define HE4 reference ranges within a Chinese study population and found the norm to be between 29.30 pmol/l and 68.79 pmol/l for premenopausal women and between 35.96 pmol/l and 114.43 pmol/l for postmenopausal women [9].

In contrast, Molina et al. defined the reference range for groups of pre and postmenopausal healthy women based on a total study population of 66 patients, as 132 pmol/l and 138 pmol/l, respectively [10]. Despite these evident associations between HE4 levels and age, diagnostic tests that involve the HE4 marker and that discriminate for menopausal status or age do not vary in either their sensitivity or specificity in the detection of ovarian cancer. In the study of Karlsen et al. that comparing RMI (Risk of Malignancy Index), ROMA, and CPH-I (Copenhagen Index, with inputs of CA125, HE4, and age), the authors found that at a sensitivity set of 75%, specificity scored 93.3%, 93.8%, and 93.2% for each test, respectively; and they concluded from this that none of the three methods is superior to any of the others [11].

### 4.2. HE4 and BMI

Our study found no association between HE4 levels and the patients' BMI. However, Bolstad et al. reported that, when comparing HE4 concentrations for BMI = 20, there was a 5% decrease in the levels with a BMI of =25 and a 10% decrease with a BMI of =30 [7]. The significance of this finding remains unknown.

### 4.3. HE4 and Smoking and Other Conditions

Apart from age, our study points to another factor that correlates with HE4 levels, namely smoking. The mechanism remains unclear. We may hypothesize that the HE4 protein participates in the body's immunological response to smoking, because chronic inflammation of smokers' pulmonary tissues leads to an overexpression of the HE4 protein. The study of Bolstad et al. goes a step further and showed that the correlation is stronger in young patients [7]. However, our study population was too small to draw any further conclusions.

It should be mentioned that other authors have reported that HE4 protein levels also correlate with chronic heart failure, where the protein level is an independent prognostic factor that determines the risks of exacerbation and death. In the same study, the authors reported a positive correlation between HE4 and other parameters, such as creatinine, NT-proBNP, galectin-3, and troponin T [12].

### 4.4. HE4 and Creatinine

Our study found no association between HE4 and creatinine. But when performing multivariate analyses, it turns out that this relationship exists if we take into account age and smoking. Our study clearly shows that age and smoking are factors that strongly affect the HE4 value. However, all our patients had creatinine levels within the reference range since those with kidney disease were excluded from the study. In turn, Bolstad has analyzed for any association with creatinine levels beyond the reference range and found a rising HE4 concentrations, which our clinical observations also confirm [7]. The reason is most probably that the impaired excretion of HE4 protein in the dysfunctional kidney leads to an accumulation of HE4 in the body. That the degree of kidney failure is related to rising HE4 concentrations is further supported by a study published in 2015, which revealed there were high HE4 concentrations in cases of kidney failure [13]. HE4 protein concentrations were high enough to invalidate differential diagnostic tests for ovarian tumors, and this fact needs to be considered when interpreting high HE4 scores. This fact also poses an important question about whether creatinine levels should be included in the diagnostic algorithm that utilizes HE4 concentrations. An answer is offered by the study of Kappelmayer et al. which showed that the logistic regression model involving CA125, HE4, and eGFR has no superior sensitivity in the detection of ovarian cancer [14]. The reason for this may be the fact that ovarian cancer diagnosis is usually undertaken in patients who have no kidney disease. In such cases, as showed by our study, normal creatinine values will have no effect on HE4 concentrations; hence, there will be no effect on ROMA and its diagnostic value.

### 4.5. HE4 and Pregnancy

In 2014, Hallamaa et al. analysed HE4 levels in a sample of 20 patients who underwent FSH treatment during in vitro fertilization [15]. He found that the HE4 levels were gonadotropin-independent. In turn, in 2015, Kanninen et al. found that among a vast array of markers, it was HE4 jointly with IL-13 that was the best predictive test for preterm labor. Compared with patients who underwent in vitro fertilization and had labor at term, those with relatively lower HE4 levels at approximately the 10th day after embryo transfer were at risk of preterm labor [16].

Our study has attempted to define the HE4 norm for healthy pregnant women for each trimester. The reference ranges we established may be of use in the detection of ovarian malignancy and in minimizing unnecessary surgical interventions that pose risks to both the mother and fetus. Although we observed no difference in the HE4 levels between the first and second trimesters, we did reveal statistically significant differences when each was compared with the third trimester (I and III, II and III). Moore et al., in a group of 67 patients, found no difference between trimesters I and II and trimesters I and III [8]. A study similar to Moore et al.'s from 2015 followed 26 pregnant patients through only their first and second trimesters and found no differences in HE4 concentrations [17]. This is in line with our findings, since the study excluded third trimester pregnancies during which we showed that HE4 rises significantly [18, 19]. What lies behind this rise in third trimester concentration levels remains unknown. Further studies on HE4 function are needed to better understand the differences in HE4 concentrations during the various stages of pregnancy and in cases of benign, borderline, or malignant adnexal changes. The use of the HE4 marker is of great diagnostic value but requires further study involving a long observational period of healthy women that would eventually develop cancer of the ovary, salpinx, or peritoneum. We express the hope that the HE4 marker will be applicable in ovarian cancer screening, which is of utmost importance to enhance the early detection rate and to allow for more effective treatment.

## 5. Conclusions

1. HE4 concentration is lowest during the first trimester, which is also the time when most tumors concurrent with pregnancy are detected. In such settings, it is crucial to bear in mind that the HE4 marker is elevated, together with the duration of the pregnancy irrespective of adnexal changes. Thus, physiological rising in HE4 levels must not be interpreted as an indication for surgical treatment
2. By defining the normal range of the HE4 protein serum level, our study makes it possible to properly interpret measurements of the marker. This can result in earlier and more efficient diagnosis of ovarian malignancy
3. We found no correlation between HE4 levels and BMI. However, we did find a positive correlation for HE4 levels and smoking and for HE4 levels and age
4. We found no correlation between HE4 concentrations and creatinine levels within the reference range. But if we take into account age and smoking in multivariate analysis, there is a correlation

## Figures and Tables

**Table 1:** Study group characteristics.

Group	All		Premenopausal		Postmenopausal	
	Total of patients (*n*)	Median age (range) (years)	*n*	Median age (range) (years)	*n*	Median age (range) (years)
Healthy women	265	48 (17-78)	157	38 (17-54)	108	59 (45-78)
Women with a benign tumor	195	43 (16-83)	139	37 (16-53)	56	60 (49-83)
Pregnant women	157	30 (18-46)	—			

**Table 2:** HE4 in healthy patients and in those with an ovarian benign tumor by age decades.

Age category (years)	Number of patients	Patients free of adnexal changes		Patients with benign adnexal changes	
		Median (pmol/l)	Range (min–max) (pmol/l)	Median (pmol/l)	Range (min–max) (pmol/l)
16-19	11	42.0	43 – 60	48	34 - 67
20-29	75	40.5	15 – 61	52.0	33 - 94
30-39	80	45.0	32 – 79	54.0	31 - 212
40-49	107	48.0	24 – 92	53.0	25 - 95
50-59	107	51.0	24 – 118	58.0	41 - 101
60-69	63	52.0	40 – 120	67.0	33 - 120
70-79	15	62.0	48 – 188	96.5	44 - 209
80-83	2	—	—	119.5	98 - 141

**Table 3:** Reference range of HE4 for healthy patients and for the extended group that includes those with benign adnexal changes.

Studied group	Menopausal status	Median (pmol/l)	Number of patients	HE4 norm (pmol/l) (CL95%)
Healthy women	All	48	265	33-85
Healthy women	Pre	44	157	15-73
Healthy women	Post	53	108	38-93
Healthy+benign conditions	All	49.5	460	33-91.5
Healthy+benign conditions	Pre	40	296	32-79
Healthy+benign conditions	Post	55	164	40-117
Patients with endometrial cysts	All	52	72	35-83
Patients with cystadenomas	All	52	51	34-119
Patients with dermoid cysts	All	53	39	33-94
Patients with serous cysts	All	51	33	37-79

**Table 4:** Association of HE4 levels with BMI, creatinine, and smoking for the healthy and the benign tumor group.

	*p*	Spearman's *r*
HE4 & BMI	0.294	0.081
HE4 & creatinine	0.997	0.001
HE4 & smoking	*0.001*	0.375

**Table 5:** Generalized linear model. Age, BMI, creatinine, and smoking used for determination of different HE4 levels for the healthy and the benign tumor group.

Effect	HE4—parameter estimates Distribution—normal Link function—identity					
	Estimate	Standard error	Wald stat.	Upper CL 95%	Lower CL 95%	*p*
Age	0.408	0.160	6.442	0.093	0.723	0.011
BMI	0.107	0.312	0.118	-0.505	0.720	0.730
Creatinine	28.557	11.455	6.214	6.104	51.010	0.012
Smoking	4.809	1.610	8.919	1.653	7.966	0.002

**Table 6:** The results of serum HE4 levels by pregnancy trimester.

Trimester	Median (pmol/l)	Range (min–max) (pmol/l)	Norm (pmol/l) (CL95%)
I	40	28 – 69	30-55
II	43	15 – 80	15-80
III	53	30 – 117	31-106

## Data Availability

The data used to support the findings of this study are available from the corresponding author upon request.
